# Supplementary material for: Are carbon emissions trading and green financial instruments synergistic? -Comprehensive quantitative research based on content analysis
Source: PLoS One. 2024 Mar 7;19(3):e0298601. doi: 10.1371/journal.pone.0298601 (PMC10919601; doi:10.1371/journal.pone.0298601)
Supplement: S1 Data — (ZIP) [file pone.0298601.s001.zip › Data/地级市Z_F工作报告.zip!/╜¡╦╒/╗┤░▓╩╨/└┤╫╘╣1⁄2╓┌║┼í╛┬φ┐╦╩2╛▌═°í┐_2014.docx]

各位代表：

现在，我代表淮安市人民政府向大会报告工作，请予审议，并请各位政协委员和其他列席人员提出意见。

2013年工作回顾

过去的一年，我们在省委省政府和市委的正确领导下，团结依靠全市人民，深入贯彻党的十八大和十八届三中全会精神，始终牢记习总书记把周总理家乡建设好的殷切嘱托，围绕市委六届三次全会确立的“两大目标”，全力聚焦“五大突破”，大力实施“十大行动计划”、“十项重点工程”，全市经济社会保持平稳健康发展，呈现出“稳中有进、质态提升、后劲增强、民生改善”的向好态势，实现了新一届政府工作的良好开局。一是综合经济实力迈上新台阶。预计地区生产总值突破2000亿元、达到2160亿元，增长12%，人均超过7200美元；规模以上工业增加值、高新技术产业产值、建筑业产值跃上千亿元台阶，分别达1134亿元、1130亿元和1032亿元。主要指标增幅继续位居全省前列，实现公共财政预算收入271.4亿元，增长16.2%，税收占比达81.6%；规模以上固定资产投资1453亿元，增长22.1%；金融机构本外币存贷款余额分别达1737亿元和1398亿元，增长14.2%和17.4%；社会消费品零售总额721亿元，增长13.9%；城乡居民人均收入分别达25456元和11045元，增长10.7%和12.3%。二是一批重点工程项目实现重大突破。连淮扬镇铁路正式立项，徐宿淮盐铁路有望尽快获批，淮安向高铁时代迈出关键一步，区域性综合交通枢纽地位基本确立；市盐化新材料产业园发展得到省政府个性化政策扶持，获批国家火炬计划特色产业基地，总投资15亿美元的实联化工纯碱项目竣工投产，将助推“中国新盐都”加快建设；白马湖作为全省唯一项目被列入国家“江河湖泊生态环境保护专项”投资计划，省运会场馆基本建成，里运河文化长廊、金融中心、科教产业园全面启动建设。全市有17个项目列入省政府2014年200个重大项目实施计划。这些都为我市全局及长远发展增创了新的优势。三是全面小康社会建设取得阶段性成果。主攻薄弱环节，补齐指标短板，全社会研发投入占地区生产总值比重、城市化率分别提高到1.5%和55%，城镇劳动保障三大保险综合覆盖面达95%以上，以市为单位达到省定2003版全面小康标准，比省定时间提前4年，清河、清浦、洪泽、金湖、盱眙顺利通过验收，为建设更高水平全面小康社会奠定了坚实基础。

过去的一年，我们重点抓了五个方面工作：

突出产业转型升级和科技创新驱动，经济发展扩量提质。工业经济实现新提升。培育了一批新的增长点，净增规模以上工业企业300户，新开工投资超亿元项目153个、新竣工103个，天士力生物医药产业园、中立方电子薄膜等15个投资超10亿元重大项目竣工投产，完成规模以上工业投资804亿元，增长22.4%。企业运行质效进一步提高，规模以上工业增加值增长13.5%，实现销售4758亿元、利税413亿元、利润219亿元，分别增长20.2%、19.4%和21.8%，新增销售超百亿元企业1户。“4+2”优势特色产业加速向高端攀升，盐化新材料产业呈现出企业规模化和集聚集群发展态势，电子信息产业正由电子元器件加工向整机生产跨越、由软件开发向信息服务延伸。现代农业取得新进展。突出龙头企业带动、新型主体培育和产业基地建设，实施投资超3000万元项目58个，新增销售超亿元龙头企业14户，组建市级以上农民专业合作社示范社404个，培育家庭农场353个，获批建设江苏淮安国家农业科技园区，淮阴区创成国家农业产业化示范基地。注重品牌培育，新注册“淮阴黑猪”等地理标志商标33件，总量全省第一、全国第四。着力夯实农业生产基础，完成水利投入33.7亿元，新建高标准农田34万亩，农机推广和粮食收购工作得到加强，粮食生产实现十连增。服务业发展迈出新步伐。开工建设中国移动呼叫中心等一批重大项目，完成规模以上经营性服务业投资249亿元，增长21.3%。29家市级以上现代服务业集聚区快速发展，综合大市场建设加快推进，淮安现代商务集聚区获批全省首批服务业综合改革试点区。周恩来故里旅游景区加快建设，漕运博物馆创成4A级景区。引进苏州银行、广发银行等10家金融机构，村镇银行实现涉农县区全覆盖。服务业增加值达900亿元，增长13.3%，占地区生产总值比重提高1个百分点。创新能力得到新增强。产学研协同创新势头强劲，引进中国科学院、东南大学等15家高校院所设立研发机构和研究生培养基地，成立苏北首家工业研究院，建成国家级盐化工产品质检中心，与北京中关村、武汉东湖国家自主创新示范区签订合作协议。企业自主创新能力显著增强，实施科技创新券制度，新建市级以上企业“两站三中心”86个，新增省级高新技术企业57家、累计达147家，新获批国家星火计划项目27个，高新技术产业产值占规模以上工业产值比重达24%。人才引进和科技招商成效明显，引进国家“千人计划”人才11名、省“双创计划”人才16名，带动燃气发动机等一批高科技项目落户。荣获全国科技进步先进市称号，区域创新能力在全国地级市排名上升至第51位。

注重激发内在活力和集聚资源要素，改革开放深入推进。各项改革不断深化。完善政务服务管理体制，全面整合公共服务和公共资源交易平台，积极筹建新的市政务服务中心。突出简政放权，清理下放行政许可10项、非许可审批6项，将61个事项由前置审批转为后置审批。实施第五轮行政提速，对行政审批流程进行再造，平均审批环节由5个缩减到3.2个。积极推进金湖县农村综合改革试点，探索创新土地利用管理制度。扎实做好县级公立医院综合改革试点，医药卫生体制改革进一步深化。总结推广马坝镇扩权强镇改革试点经验，经济发达镇行政管理体制改革有序推进。招商引资形成新格局。在巩固闽资、浙资高地的基础上，北上广深和环渤海湾等区域招商引资取得突破，北京首创、上海国际、中兴通讯、青岛海尔等一批知名企业相继落户；在主攻台资、港资的同时，积极拓展欧美日韩招商，新批项目14个。新引进投资超亿元或超3000万美元项目231个，其中超10亿元或超9000万美元项目25个。外资利用质态得到提升，实际利用外资13亿美元，开工项目到账外资占比达61.5%，提高了28.3个百分点。产业链项目招引取得重大进展，中兴4G智慧产业园、天地龙智能电网等一批投资超30亿元重大项目正式签约，新世代半导体、惠浦OLED液晶显示屏等一批投资超5亿美元外资项目成功引进，富士康智能电视、奔驰系列商用客车等一批终端产品项目相继落户，将带动主导产业转型升级，助推新兴产业集群发展。台资高地加速崛起。成功举办第八届台商论坛、“台湾·淮安周”等系列活动，淮安在岛内影响不断扩大，再次荣获台商投资“极力推荐城市”称号。

新落户耐斯、江兴锻压等知名台企，引进京永自行车、丰华生物医药等产业项目，实际利用台资5.8亿美元，居全省第二，台资集聚效应更加凸显。园区载体全面提升。综合保税区正式封关运行，启动规划建设12个国别园区，盱眙凹土科技园获批省级产业集聚示范区。在全省开发区综合考评中，6家省级以上开发区实现进位，淮安经济技术开发区排名升至第22位，涟水、金湖经济开发区首次进入全省第一板块。

重抓中心城市建设和基础设施配套，城乡统筹步伐加快。中心城市建设全面推进。投入890多亿元，实施867个城建项目，“四馆”、大剧院等基本竣工，健康东路整治及大同路中段建设、水门桥改造等全面完成，雨润新天地、茂业百货、力宝广场等项目进展顺利，公共自行车一期工程和市老年公寓建成投运，现代有轨电车1号线启动建设，城市功能进一步增强。全面启动城市环境综合整治，市容市貌不断提升。强化四个县城对中心城市的支撑作用，加快建设富有特色的中等城市。房地产市场平稳发展。重大基础设施逐步完善。宿淮铁路全面建成，高铁规划建设前期工作基本完成。一类航空口岸开放列入国家年度审理计划，淮安涟水机场通航城市增加到16个。205国道淮安西绕城段基本建成，金马高速建成通车，实现了县县通高速。南水北调东线一期工程建成通水，入江水道整治有序推进，盐河航道整治全面完成。小城镇和新农村加快建设。完成15个乡镇、80个村庄规划修编，着力打造层次分明、特色鲜明的“4820”市域小城镇体系。加大镇村基础设施建设力度，新改建农村公路636公里，新建区域水厂2座，新增区域供水乡镇8个。加强新型农村社区建设，完成9135个村庄环境整治，新创三星级康居乡村16个，码头镇码头村和塔集镇陆河村被确定为全国美丽乡村创建试点村。加快产镇融合步伐，乡镇工业集中区实现开票销售484亿元，新入驻投资超3000万元项目244个。生态文明建设成效明显。科学划定生态红线，扎实推进国家生态市创建，投入6.6亿元建成一批乡镇污水、垃圾等处理设施，新增成片林4.3万亩，金湖、清浦在苏北率先创成省级生态县区并通过国家级技术评估，新增14个国家级生态乡镇和286个市级以上生态村。加强国家低碳试点城市建设，淮安经济技术开发区获批国家级循环化改造试点园区，生态新城创成苏北唯一的省建筑节能与绿色建筑示范区。PM2.5监测实现全覆盖，节能减排完成省定目标。

提升公共服务能力和民生保障水平，群众生活持续改善。社会事业加快发展。全市财政性教育投入超过70亿元，以市为单位实现区域教育基本现代化，义务教育质量监测成绩进入全省前列，高考二本以上录取率苏北第一。获批筹建江苏护理职业技术学院，新增3所国家中等职业教育改革发展示范学校，高校人才培养质量和产学研合作水平不断提升。组织开展千名淮医进社区等惠民活动，新创13个省级示范乡镇卫生院，市中医院创成三甲医院。大运河淮安段申遗顺利通过验收，首获全国优秀儿童文学奖、全国戏剧文化奖和省文华大奖。精心组织省运会筹备工作，成功承办一批全国和省级赛事。人口和计划生育公共服务体系不断完善，成为全国创建幸福家庭活动试点市。社会保障不断加强。企业退休人员基本养老金上调10%，城镇居民医保补助标准提高到人均320元，城乡居民养老保险、城镇居民医疗保险和新农合参保率分别达97%、95%和99.9%。加大扶贫开发力度，投入扶贫资金5.7亿元，14.8万农村低收入人口实现脱贫。十件实事全面完成。投入35.8亿元，完成十大类20项民生实事。新建40所幼儿园，全面完成中小学校安工程三年任务；新建10所惠民示范医院，实行免费婚检和孕检；市区新建10个公交站场，新增80辆新能源公交车和260辆出租车，新开通镇村公交乡镇17个；城乡低保标准分别提高到每人每月410元和290元，城乡居民大病保险报销比例超过50%，落实残疾人庇护性劳动项目45个，5.13万户城乡低保家庭享受免费收看有线电视；超额完成保障性住房建设、农贸市场改造、农村金融服务体系建设等任务。

强化政府自身建设和社会管理创新，履职能力不断增强。更加注重统筹谋划。按照党的十八大“五位一体”总体布局、省委省政府“八项工程”工作部署和市委跨越发展目标要求，分别制定“十大行动计划”和“五大突破”实施方案，明确了未来3-5年的工作目标和重点举措。切实把握新时期淮安发展的新特征新要求，坚持规划引领，推进全域规划、多规融合，完成市域主体功能区等综合性规划，加快构建“一区两片四轴”总体空间格局，高水平编制里运河文化长廊、白马湖保护开发等一批专项规划，努力以科学规划提升决策水平、增强投资信心、引领跨越发展。更加注重政策创新。出台扶持民营经济发展“十八条意见”，在全省率先实行工商登记“先照后证”和注册资本“零首付”，加大初次创业补贴、小额贷款贴息等扶持力度，推动新一轮全民创业、淮商崛起，新发展私营企业1.16万户，新增注册资本351.2亿元。制定加强市区土地经营管理“五统一”意见和“飞地”政策，推行用地指标市场交易，促进土地资源节约集约利用。争取中国人民银行南京分行出台专项金融支持政策，实施科技和小微企业小额贷款保证保险，信贷融资实现较快增长。更加注重重点突破。立足长远发展和群众期盼，以铁路规划建设等“十项重点工程”为抓手，强化统筹谋划和向上争取，有效推动重大基础设施、城市功能项目和园区载体建设取得突破性进展。突出加快县域经济发展，实行“一县一题”专项扶持，支持淮阴区创建国家农业科技园区，推动盱眙宁淮新兴科技产业园和涟水盐河产业带规划建设，淮安古城保护开发、洪泽湖旅游开发及金湖“一主四辅”城镇体系规划建设等取得重要进展。完善重大项目推进机制，加大市直部门招商考核力度，采取季观摩、双月谈、月例会等方式，强化督查会办，项目建设的速度和效率显著提高。更加注重依法行政。大力推行行政权力网上公开透明运行，在苏北率先通过省法治政府建设阶段性目标考核验收。认真落实中央和省、市委关于改进工作作风、密切联系群众的各项规定，建立健全长效机制，切实规范行政行为，政府自身建设不断加强。更加注重协调推进。深入开展大接访、法治惠民活动，重大事项全部实现社会稳定风险评估，金盾护航、网格化社会管理、十大维权中心和十大警务中心建设等创新举措不断完善。进一步健全公共安全和应急管理机制，预防犯罪、消防检查和食品安全等工作扎实有效，安全生产形势保持平稳。第五届社区居委会和第十届村委会换届选举工作顺利完成。双拥“三就”工作经验在全国推广。新增国际友城、友好交流城市10个，国际交流合作层次不断提升。援疆工作成绩显著，十大援建项目进展顺利。统计、审计、国资、物价、民防、供电、供销、仲裁、海关、检验检疫、机关事务、民族宗教、广播电视、妇女儿童、青少年、老龄、关心下一代、红十字、老区开发、地震、气象、档案、地方志等工作都取得新的业绩。

各位代表，过去的一年，我们沉着应对复杂多变的宏观经济形势，全力克服不断增大的经济下行压力，突出谋划布局、主动调整、打牢基础、加快发展，取得了来之不易的成绩。这是省委省政府和市委正确领导的结果，是市人大、市政协及社会各界关心支持的结果，是全市人民团结奋斗的结果。在此，我代表市人民政府，向全市广大干部群众，向全体人大代表和政协委员，向各民主党派、工商联、无党派人士、各人民团体和离退休老同志，向各驻淮单位及部队指战员，向所有关心、支持和参与淮安建设发展的企业家和海内外朋友，表示衷心的感谢并致以崇高的敬意!

在总结成绩的同时，我们也清醒地认识到，全市经济社会发展中还存在不少矛盾和问题：部分经济指标增幅回落，少数指标未能达到预期目标，进位争先压力不断加大；产业集聚程度不够高，科技支撑能力还不强，转型升级任务还很艰巨；县域经济发展不够充分，二元体制矛盾比较突出，统筹城乡发展步伐有待加快；去年下半年以来空气污染指数时有反弹，保护生态环境的措施亟待加强；深化改革、保障民生的力度与人民群众的期望还存在一定差距，行政效能、服务意识仍需提高。对此，我们将在今后的工作中切实加以解决。

2014年目标任务

今年，我市将开启全面深化改革、建设更高水平全面小康社会的新征程。综观当前宏观经济形势，仍然较为严峻复杂，国际上以刺激政策带动的反弹周期趋于结束，新的增长动力源尚不明朗；国内经济正处于“三期叠加”的关键阶段，一些长期积累的矛盾将进一步凸显。同时，我们也看到，世界经济缓慢复苏的趋势没有改变，我国经济发展长期向好的基本面没有改变，特别是党的十八届三中全会作出的战略部署所释放的改革红利，省委省政府支持苏北全面小康社会建设系列政策所形成的叠加优势，将为我市创造良好的发展环境。尤为重要的是，经过多年发展，淮安以优势特色产业集群发展为主导的新型工业化进程、以中心城市建设为引领的城乡发展一体化进程、以台资集聚高地崛起为特征的对外开放进程和以国家生态市创建为抓手的生态文明建设进程不断加快，内在活力和竞争优势日益增强。只要我们把握大局、看清走势，积极应对、负重奋进，就一定能在日趋激烈的竞争中巩固提升现有位次，保持持续发展的良好态势，在新起点上实现新跨越。

今年政府工作的总体思路是：全面贯彻党的十八大、十八届三中全会和中央、省经济工作会议精神，紧紧围绕省“八项工程”总体部署，按照市第六次党代会第三次会议要求，坚持稳中求进的工作总基调，紧扣“两大目标”，实施“六大战略”，推进“五大突破”，重抓“十项重点工程”，切实以改革增活力，以投入保增长，以开放聚要素，以创新促转型，以发展惠民生，努力实现“经济持续增长、发展持续转型、城乡持续提质、民生持续改善”，确保以县为单位达到省定2003版全面小康指标体系要求。

今年全市经济社会发展的主要预期目标是：地区生产总值增长11%左右，公共财政预算收入增长12%左右，规模以上固定资产投资增长20%，社会消费品零售总额增长12%左右，城乡居民人均收入增长10%，城镇登记失业率控制在4%以内，居民消费价格指数不高于省均水平，节能减排完成省定任务。

实现今年的奋斗目标，我们将重点抓好六个方面的工作：

(一)坚持改革创新发展，增创跨越争先新优势。着眼发展出题目、改革做文章、创新求突破，努力以改革创新抢占先机、争创优势、赢得发展。

全面深化各项改革。准确把握中央和省全面深化改革的总体部署，按照市委《实施意见》的要求，统筹谋划，分类推进，着力构建具有时代特征和淮安特色的改革体系。深化以市场为导向的经济体制改革，放宽市场准入，优化资源配置，加快转变经济发展方式；深化以人为核心的新型城镇化改革，着力破除体制机制障碍，加快城乡发展一体化步伐；深化以增创竞争优势为目的的科技体制改革，加快产学研协同创新步伐，促进产业转型升级；深化以转变政府职能为重点的行政管理体制改革，着力做好减少审批、整合职能、扶持发展三篇文章，进一步提升服务效能；深化以改善民生为根本的社会治理体制改革，在公共服务、社会事业、民生保障等领域先行先试，不断提高人民群众的幸福指数。今年，重点在三大领域取得突破：一是围绕加快中心城市建设和新型城镇化发展，推进户籍制度、扩权强镇、基本公共服务均衡化、农村经营管理体制、土地利用管理制度等配套改革，争取成为省级新型城镇化综合配套改革试点市。二是围绕强化金融服务，积极推进金融改革，进一步优化金融生态环境，吸引更多国内外银行来淮设立分支机构，积极支持创投风投、私募股权基金发展，鼓励民营资本组建农村小贷公司等金融组织，支持企业扩大直接融资，力争4户企业在境内外上市挂牌。三是围绕建设服务型政府，加快推进行政管理体制改革，加大审批事项的“减、转、放、免”力度；统筹推进政府机构与事业单位分类改革，不断扩大政府社会管理职能委托、授权试点，让各类主体的发展活力充分迸发。

着力提升创新能力。大力实施创新驱动战略，加快国家创新型试点城市建设。强化企业创新主体地位，发挥科技创新券引导作用，促进企业加大研发投入，培育创新型领军企业5家，新增省级高新技术企业25家以上，高新技术产业产值占规模以上工业产值比重超过25%。加强产学研协同创新，推进与知名高校院所和科技型企业合作，积极鼓励本地高校加强科技创新，加快凹土等10个重大产业研发机构建设，新建市级以上企业“两站三中心”140个。加速高端资源集聚，深化与北京中关村、上海张江、武汉东湖的战略合作，着力打造智慧产业谷，建成江苏省大学毕业生苏北人才市场，招引高科技项目100个、前瞻技术应用项目10个，引进“千人计划”人才10名、“双创计划”人才16名以上，让科技创新真正成为淮安跨越发展的第一驱动力。

不断优化发展环境。按照“一个中心依托、一套制度支持、一个品牌指引”的思路，切实加强政务服务体系建设，努力以高效服务赢得快速发展。建成市政务服务中心，实现“三集中、三到位”，打造全国一流的政务服务平台。推进新一轮行政提速，切实优化并联审批流程，全面实施“四证联办”、“四审联动”和模拟审批制度，着力解决多头审批、重复审批、超时审批等问题。不断丰富101%服务品牌内涵，从生产经营、生活配套、权益保障等方面进一步细化措施，健全重大项目帮办服务及协调推进机制，为各类市场主体提供更加便捷高效服务，真正使淮安成为资源要素集聚的沃土，成为企业家寻梦追梦圆梦的乐园。

(二)坚持转型升级发展，培育壮大优势特色产业。全力实施工业第一方略，推动先进制造业与现代服务业互动并进。实现规模以上工业增加值1260亿元，增长12.5%，服务业增加值突破千亿元。

推动先进制造业做大做强。重抓产业转型升级，按照一个产业制定一项规划、落实一套政策、组建一个班子、出台一个招商指导意见、主攻一批重大项目的要求，大力开展“4+2”优势特色产业突破年活动。实施井神盐业盐碱一体化、方圆化工等项目，带动盐化新材料产业依托“两碱”向高端化学制品、生物材料方向拓展；推进杰亚精密铸造等项目建设，重点延伸特钢产业链，加快向装备机械方向发展；推进富士康智能电视、新世代半导体等项目，促进电子信息产业拉长链条、做大规模、提升层次；以旺旺食品工业园四期、双汇5.5万吨肉制品加工等项目为龙头，推动食品产业规模化、品牌化发展；依托敏实、欧陆等企业，加快打造全国重要的新能源汽车及零部件研发生产基地。主攻重大项目建设，强化投资强度、开票销售、入库税收、工业用电、进项税抵扣等关键指标考核，务必做到开工建设一批、竣工投产一批、谋划储备一批，确保新开工投资超亿元项目150个、竣工投产设备投资超千万元项目100个，完成规模以上工业投资增长20%以上，新增规模以上企业200户。聚焦骨干企业培育，深化“一企一策”，整合扶持资金，加大要素保障、市场开拓、战略重组等支持力度，促进企业向规模化、集团化方向发展。

推动现代服务业做精做特。进一步优化布局、提高质态、集聚发展，努力打造区域性现代服务业高地。大力发展服务业集聚区，加快淮安现代商务集聚区、综合大市场、电子商务园等建设，实施投资超亿元经营性服务业项目150个，完成投资300亿元，增长20%。加快发展新兴服务业，推进中兴4G智慧产业园、中国移动呼叫中心等项目建设，提升“一核四特”软件园区承载功能，着力打造苏北软件和信息服务产业第一高地。积极培育现代金融、电子商务、总部经济和养老服务等新兴业态，着力发展现代物流、物联网等生产性服务业，加快融入长三角物流信息一体化平台。扶持发展现代旅游业，高标准建设洪泽湖旅游综合体、白马湖休闲度假区、西游记乐园、古淮河生态文化园，周恩来故里旅游景区力争创成5A级景区。

推动开发园区提档升级。围绕打造经济发展主平台、对外开放主阵地、产城融合主载体，不断增强开发园区综合功能。以增强科技创新能力和产业集群发展为主攻方向，加快提升国家和省级开发区建设水平，高标准建设科教产业园等创新创业核心区，推动淮安高新区、市盐化新材料产业园区分别创建国家级和省级高新区，淮安经济技术开发区、洪泽经济开发区创成省创新型开发区。按照“五个一”的要求，积极推行“飞地”政策，着力打造一批特色产业园区，推动每个县区发展壮大1-2个百亿级特色产业。积极培育特殊功能园区，完善综合保税区、空港产业园等园区功能配套，加快建设12个国别园区，着力打造经济发展新的增长极和城市扩张新的载体。

(三)坚持开放引领发展，全面增强要素集聚能力。强化开放型经济龙头作用，进一步拓展空间、提升质效，不断创造区域发展新的比较优势和竞争优势。力争实际利用外资16亿美元，进出口总额达到40亿美元。

争创台资高地新优势。积极创建国家级台资产业转移集聚和服务示范区，突出招引终端产品、龙头企业和功能性机构，加快台资集聚步伐。打造台商投资高地，坚持集群承接、沿链引进，深入台湾本岛开展系列经贸活动，精心组织珠三角、长三角等台资密集区专题招商，新引进投资超千万美元台资项目30个，实际利用台资6.5亿美元。打造台资产业高地，支持在淮台企拓展市场空间、加大技改投入、培育自主品牌，带动产业链向上下游延伸。打造服务台商高地，加强特色服务、生活配套等方面的设施建设，切实为台商营造家的氛围。打造淮台交流高地，积极对接两岸企业家峰会等高端平台，放大台商论坛品牌效应，纵深推动县区经济开发区与台湾科技园共建园区，使淮安在两岸交流合作中作出更大的贡献。

拓宽对外开放新领域。突出对外招商能力建设，拓宽境外招商渠道，开展上海、北京、广东等外资密集区驻点招商，加强与外国商会、驻华使领馆等联系，力争引进投资超3000万美元项目40个。加大欧美日韩专题招商力度，新引进项目30个。扩大外资利用范围，引进一批金融、教育、文化、医疗等项目，服务业利用外资占比达20%以上。拓展对外经贸合作空间，支持企业巩固欧美日等传统市场，积极开拓南美、中东、非洲等新兴市场，培育进出口新增长点企业100户，新增进出口超亿美元企业2户以上。坚持对外交往与经贸合作相结合，新增国际友城、友好交流城市6个以上。

提升区域合作新水平。抢抓上海自贸区建设、国有企业改革等重大机遇，加强与大集团大企业的合作，加快引进资本、技术等高端资源。在巩固苏南、闽浙等地招商的基础上，强化环渤海湾和珠三角等区域招商，着力引进一批产业链龙头项目。全面推进宁淮合作，加快交通、医疗、教育等同城化进程，推进宁淮现代服务业集聚区建设，争取获批省级南北共建园区。全年新招引投资超亿元内资项目210个，其中超10亿元项目20个。积极策应沿海开发战略，推进与连云港港口的无缝对接，带动盐河产业带加快建设。全力争取淮河经济带规划上升为国家区域发展战略。继续做好援疆项目建设、人才交流等工作。

(四)坚持城乡统筹发展，促进县域经济更大突破。完善“一县一题”帮扶机制，加快富民壮村强乡镇步伐，实现县区主要指标增幅在全省进位争先。

大力发展现代农业。强化科技、项目、品牌支撑，重点实施四项工程，加快农业现代化进程。实施新型经营主体培育工程，引进投资超3000万元项目20个，新增省级以上龙头企业8户，创建市级以上农民专业合作社示范社100个，发展家庭农场300家。实施产业质效提升工程，引进推广农业新技术30项、新品种40个，建设农产品质量追溯管理平台；发挥地理标志证明商标的品牌效应，做大精品蔬菜、特色水产等百亿级产业，获批1个国家级和5个省级“一村一品”示范乡镇。实施园区提升工程，加快国家农业科技园区建设，提升省级农业产业园区发展水平，培育3-4个农业精品园区，支持金湖、洪泽争创国家现代农业示范区。实施基础设施配套工程，完成农村水利投入8.5亿元，改造大型灌区6个，新建高标准农田25万亩。统筹推进黄河故道综合开发，切实加大刘老庄和灌溉总渠以北片区扶贫开发力度。进一步强化耕地保护，确保粮食稳产增产。

切实壮大县乡工业。针对县域工业低小散状况，加快园区功能配套和项目建设，促进产业集聚集群发展，强化县域经济跨越发展的产业支撑。大力培育县域特色产业，主攻产业链项目招引，加快打造一批百亿级特色产业基地。加强乡镇工业园区建设，重抓30个重点工业集中区扩量提质，考评认定一批市级特色工业园区，支持马坝、施河等工业集中区创建省级经济开发区，引导新启动的村级创业点进驻乡镇工业集中区，建成标准化厂房120万平方米，新增规模以上企业60户。深入开展全民创业、淮商崛起行动，全面落实民营经济发展“十八条意见”，培育壮大本土企业家队伍，引导中小企业向“专精特新”方向发展，扶持发展一批旗舰型民营企业。新培育私营企业1万户、个体工商户2.5万户，努力让淮安大地创业大潮奔流涌动、淮商新星竞相涌现。

加快推进城镇建设。坚持以中心城市为依托，以县城、重点中心镇和特色小城镇为载体，加快构建级配合理、功能互补、各具特色的市域城镇体系。按照“一县一特”要求，支持四个县城立足自身优势，进一步做大规模、做强功能、做优环境，鼓励具备条件的县加快撤县建市步伐。重点突破4个县域次中心镇和8个重点中心镇，加快打造一批工业商贸强镇、文化旅游名镇和绿色生态宜居镇。通过规划调控、产业带动和市场引导，积极稳妥推进撤乡并镇。坚持规模适中、组团发展，合理规划兴建100个新型农村社区，推动医疗、文化、交通等基本公共服务向农村延伸。

着力打造宜居环境。严守生态红线和环保底线，打好污染防治和生态创建攻坚战。铁腕整治空气污染，对电力、钢铁、石化行业的11家重点燃煤企业进行脱硫脱硝除尘改造，对全市1400多台小锅炉开展综合整治，对7户重点企业实施工业挥发性有机物治理，完善4个省级化工园区的基础供热、废水集中处理设施，加强建设工地扬尘、渣土管理，淘汰5900辆黄标车，积极引导市民低碳出行。在强化秸秆禁烧宣传引导、督促检查的同时，加大综合利用奖补力度，实施秸秆收贮、预处理等项目30个。加强国家生态市创建，以森林公园、自然湿地和农田林网为载体，加快构建城乡绿色生态系统，新增碳汇林1.2万亩；加强水资源保护和水环境治理，确保饮用水安全。力争建成2个国家级生态县区和4个省级生态县区，创成20个国家级生态乡镇，力争通过省级生态市技术评估。加快国家低碳试点城市建设，加强高能耗企业碳排放总量控制，抓好低碳交通、低碳建筑、低碳社区等示范项目建设，努力使淮安的天更蓝、水更清、地更绿，让生态真正成为淮安持续发展的“王牌”。

(五)坚持内涵特色发展，着力打造现代精品城市。按照苏北重要中心城市总体定位，重点实施八大类839个项目，完成投资1100亿元，不断提升城市功能，打造宜业宜居环境。

以规划体系的完善引领城市发展。积极策应长三角区域经济一体化和江苏沿海开发等发展战略，以打造新的功能区为重点，组织谋划新一轮城市总体规划修编，着力构建江苏新兴增长极。强化多规融合、统筹衔接，进一步完善规划体系。严格规划实施，加强城市设计，实施城市形象识别系统规划，强化城市轮廓线、标志性建筑等建设管理，切实发挥好规划的引领和调控作用。

以重点区域的开发提高城市品位。聚焦提升主城区，推进雨润新天地、金融中心等标志性建筑建设，建成苏宁广场、淮安新天地等重点项目，增强淮海中心商业区和水渡口中央商务区集聚功能。保护开发古城区，以里运河文化长廊为轴线，全面推进淮安古城、清江浦历史风貌区和码头古镇等保护开发，开工建设运河主题文化体验园、河下古镇影视基地等重点项目，加快打造淮安版的“清明上河图”。加快建设新城区，生态新城突出行政、文化、体育三大中心建设，清河新区、清浦新南城、淮阴新北城和南部工业新城重点完善基础设施，尽快形成功能齐全、环境优美、人气旺盛的城市新组团。积极改造旧城区，推进华丰装饰城、文庙三期等重点片区改造，加快西南化工区搬迁，进一步完善设施配套，改善居住环境。

以重大工程的实施增强城市功能。推进综合交通枢纽建设，启动建设连淮扬镇铁路，争取徐宿淮盐铁路获批立项，同步推进高铁枢纽规划与建设，开工建设235、346省道涟水绕城段和601省道旧铺段，争取实施淮河入海水道二期工程，加快淮安涟水机场二期扩建，力争开放国家一类航空口岸并开通淮台航线。推进市区道路通达工程，实施淮海南北路提升改造，打通漕运西路等断头路，建成淮海路盐河桥、通甫路南延等项目。基本建成现代有轨电车1号线，加快三城融合步伐。推进城市绿化美化工程，实施市区主干道以及城市出入口景观提升、宁连路绕城段绿化、白马湖湖滨森林公园等项目。推进城市水环境整治，开工建设里运河北门桥控制闸等工程，进一步完善城市给排水管网，不断提升滨河景观，彰显绿水城市风貌。

以综合长效的管理塑造城市形象。健全管理重心下移、执法集中统一、建管养分离的长效机制，集中开展城市环境“618”整治工程，实施重点区域夜景亮化和建筑立面出新，加快中欧绿色智慧试点城市和数字城管建设。推进国际化语言标识设置，让淮安更有“国际范儿”。下大力气解决交通拥堵问题，优化交通组织，严格秩序管理，整治占道经营、乱停乱放等现象，切实提高道路通畅率。规划建设一批公共停车场，新增停车泊位3500个以上，有效缓解停车难问题。坚持改善城市环境与提高市民素质相结合，积极创建全国文明城市，努力使我们的城市更文明，让市民的生活更美好。

(六)坚持民生优先发展，提高公共服务和社会治理水平。把人民对美好生活的向往作为工作方向，加大民生投入，加强社会治理，不断增进民生福祉，促进社会和谐。

大力发展社会事业。积极推进教育现代化建设，加快发展学前教育，优质均衡发展义务教育，健全现代职业教育体系，开工建设淮安开放大学，积极筹建淮安综合性大学。提升公共卫生服务能力，开工建设市眼科医院，规划建设市儿童医院，争创三甲医院1所、省示范乡镇卫生院12所。实施文化惠民工程，加快县区文化场馆、社区文化中心建设，免费开放公共文化设施，创作一批国家级文艺精品。落实计划生育政策，促进人口长期均衡发展。举全市之力办好省运会，加强场馆设施配套和服务保障，开展全民健身与省运同行活动，精心做好赛事组织，努力取得优异成绩，进一步提升城市美誉度。

加大就业扶持力度。突出抓好高校毕业生、城镇就业困难人员、农村劳动力等重点人群就业，做好政府购买公益性岗位人员招募工作，建立农民就业失业登记等制度，落实农村劳动力获证奖补政策。坚持以创业带动就业，深入开展省级创业型城市建设，完善城乡一体化创业扶持政策，开展多形式、多层次免费创业培训，完成创业培训2万人次。落实好农民自主创业小额贷款等扶持政策，新建创业孵化基地22个，扶持1.2万人创业，新增城镇就业再就业6万人以上。

加强社会治理创新。推行政社互动社会治理新模式，发挥好社会组织和基层群众自治组织作用，引导和组织非公企业参与社会治理。畅通群众诉求表达渠道，优化社会矛盾纠纷调处机制，打造阳光信访升级版，及时就地解决群众合理诉求。整合各类应急资源，加强政府应急综合指挥平台建设。深化平安淮安创建，积极构建立体式的社会治安防控体系。切实抓好安全生产，建立健全安全生产责任网、监管网和保障网。加强食品药品监管，积极营造放心的消费环境。认真做好人民武装和双拥共建工作，进一步推进军民融合深度发展。

各位代表，民生是为政之本，今年我们将继续围绕人民群众关心的热点、难点问题，投入40.1亿元，办好保障和改善民生十件实事。一是老旧小区改造，对150万平方米的老旧小区配套设施进行综合整治。二是公共交通能力提升，加大公交改革发展力度，完善市区10个公交(出租车)场站功能，新增80辆新能源公交车，更新出租车800辆以上，增加3700辆公共自行车，新改扩建12条城区道路，整治改善63条后街背巷，新改建农村公路400公里、桥梁50座。三是农村环境综合整治，坚持规划引领，对8830个村庄进行综合整治，完成6万座农村无害化户厕改造，疏浚315条县乡河道，整治1183个村庄河塘。四是农村饮用水安全，铺设区域干线管网245公里，新增通达乡镇24个，改善12.6万人饮水条件。五是农贸市场升级改造，对市区12个农贸市场进行标准化升级改造，切实提升食品安全保障水平。六是困难群体住房保障，新开工建设公共租赁住房1258套、经济适用住房432套，廉租住房租赁补贴实现应保尽保。七是改善基层办学条件，按照省定一类标准为100所农村中小学配备相关教学设备，按照省级优质标准新建40所幼儿园。八是创建惠民示范医院，新改扩建10所乡镇卫生院和社区卫生服务中心，建成市口腔医院。九是尊老爱老济困助残，为城乡年满60周岁纳入保险的居民每人每月增发10元基础养老金，城乡低保标准分别提高到450元和330元，建设10个农村“关爱驿站”和20个区域性养老服务中心，为5000户残疾人家庭进行辅助器具适配和无障碍环境改造。十是完善便民服务设施，新建“城市绿色小客厅”(游园)3.7万平方米、公共厕所54座，在137个社区建设“10分钟体育健身圈”，建成46公里健身步道、30个社区文化中心。

各位代表，开创改革开放新局面，实现跨越发展新目标，谱写好中国梦的淮安新篇章，我们深感责任重大、使命光荣。必须大力弘扬“包容天下、崛起江淮”的新时期淮安精神，积极顺应群众对政府工作求新求快求实的要求，坚持以高效廉洁为价值取向，恪尽职守，永不懈怠，加快建设人民满意的服务型政府。一是联系群众聚合力。扎实开展党的群众路线教育实践活动，突出为民务实清廉主题，坚决克服形式主义、官僚主义、享乐主义和奢靡之风，全心全意当好人民公仆。坚持将新增财力四分之三以上用于改善民生，努力提供更为完善的社会服务，下大力气解决群众最关心、反映最强烈的突出问题。切实提高政府公信力，凡是向群众和客商承诺的事情，一诺千金、说到做到，坚决不打折扣。推动各级干部下基层、接地气，和群众说掏心窝的话，为百姓做暖心窝的事，进一步密切联系群众，切实凝聚起加快发展的强大合力。二是锐意改革善创新。坚持以改革增红利、靠创新添动力，以“先行先试、善作善成”的勇气和智慧，加快破除影响科学跨越发展的思想障碍和体制弊端。处理好政府与市场的关系，创新行政管理方式，加快转变政府职能，着力打破利益藩篱，进一步简政放权、优化服务。坚持顶层设计与基层创新相结合，因地制宜、大胆实践，努力使中央和省全面深化改革的各项部署在淮安落地生根、开花结果。三是矢志发展敢担当。切实增强时不我待的使命感，提振攻坚克难的精气神，勇于在逆势中争先，善于在深水区游泳，做到谋划发展有点子、破解难题有法子、向上争取有路子，力争更多的工作在全国有亮点、在全省有位置。坚持“项目为王”的理念，促进各级干部将更多的精力花在项目上，善于谋划项目，勤于推动项目，敢于争取项目，努力做到在项目招引上一马当先，在项目审批上一路绿灯，在项目建设上一抓到底，真正使项目成为推动跨越发展的强大支撑，成为衡量干部实绩的重要标准。突出政府执行力建设，以市委市政府工作目标为依据，以提高群众满意度为追求，进一步强化督查考核，加大问责力度，狠抓工作落实，努力创造无愧于时代和人民的新业绩。四是清正廉洁树新风。严格执行中央和省、市委加强党风廉政建设的各项规定，始终将党的优良作风和严明纪律内化于心、外化于行，树立勤政廉洁政府良好形象。强化运行监督，推进政务公开，自觉接受人大及其常委会的法律监督和工作监督，主动接受政协的民主监督及社会舆论监督，让权力在阳光下运行。坚持从严治政，严格控制“三公”经费，强化行政监察和审计监督，健全惩治和预防腐败体系，努力建设勤勉尽责、作风优良的高素质公务员队伍。

各位代表，实干播种希望，拼搏创造未来。让我们紧密团结在以习近平同志为总书记的党中央周围，在省委省政府和市委的正确领导下，同心同德，开拓创新，务实奋进，为加快建设苏北重要中心城市和更高水平全面小康社会而努力奋斗!
